# Munchausen Syndrome in the Context of Liaison Psychiatry: A Case Report and a Narrative Review

**DOI:** 10.7759/cureus.54289

**Published:** 2024-02-16

**Authors:** Odete Nombora, Eva Mendes, André Oliveira, Lúcia Ribeiro

**Affiliations:** 1 Psychiatry Department, Vila Nova de Gaia/Espinho Hospital Centre, Vila Nova de Gaia, PRT; 2 Psychiatry Department, Liaison Psychiatry Unit, Vila Nova de Gaia/Espinho Hospital Center, Vila Nova de Gaia, PRT

**Keywords:** self-inflicted injury, liaison psychiatry, plastic surgery, factitious disorder, munchausen syndrome

## Abstract

Munchausen Syndrome (MS) has been widely recognized as a severe manifestation of factitious disorder, a condition where individuals intentionally fabricate or exaggerate symptoms for psychological gratification. It represents a complex diagnostic challenge due to its elusive nature and intricate relationship with various medical conditions. We present a clinical case of a 44-year-old woman observed in the context of Liaison Psychiatry, demonstrating the intricate interplay between chronic medical conditions, psychiatric factors, and the challenges in diagnosing and managing MS. The patient exhibited a history of recurrent hospitalizations, difficult-to-heal injuries, and a pronounced preference for surgical interventions. Despite diagnostic difficulties and poor therapeutic adherence, a multidisciplinary team approach involving plastic surgery, orthopedics, physical medicine, and rehabilitation, alongside Liaison Psychiatry, led to the diagnosis of MS with chronic osteomyelitis, ultimately necessitating a transtibial amputation. The case underscores the importance of early detection, a multidisciplinary approach, and the role of Liaison Psychiatry in managing MS. While early diagnosis may not alter the disease course, it can prevent unnecessary interventions and mitigate associated risks. The case also highlights the need for continuous psychiatric support and family involvement in addressing the recurrence of self-injurious behaviors. Further research is essential to enhance our understanding and develop effective treatment strategies for MS, contributing to improved diagnostic precision and overall management of this challenging psychiatric disorder.

## Introduction

Since its initial characterization in 1851, Munchausen Syndrome (MS) has been widely recognized as a severe manifestation of factitious disorder, a condition where individuals intentionally fabricate or exaggerate symptoms for psychological gratification [1-3]. Historically, factitious disorders, including MS, have posed diagnostic challenges due to their elusive nature and the blurring boundaries with other related conditions [2-5].

Factitious disorders have been recognized for centuries. In 1843, Hector Gavin (1815-1855) proposed an initial distinction between simulation and factitious symptoms. During the reconsideration of hysteria and simulation in 1908 by Joseph Babinski (1857-1932), French physician Georges Dieulafoy (1839-1911) introduced the term “pathomimie”. He enlisted his friend, French writer Paul Bourget (1852-1935), to coin a new term for describing self-induced skin lesions observed in patients [4].

The term “Munchausen Syndrome” was coined in 1951 by Richard Asher, inspired by the infamous Baron von Münchhausen, a German nobleman known for his extravagant and false narratives [4,5]. Twelve years before the Baron's death in 1785, Rudolph Erich Raspe (1737-1794) anonymously published the first edition of Baron von Münchhausen's tales. Asher adapted the name to “Munchausen”, a spelling now used in the medical literature [5]. The initial description of the syndrome focused on three main clinical presentations: abdominal, neurological, and hemorrhagic [2,4,5]. In 1967, French hematologist Jean Bernard (1907-2006) expanded the hemorrhagic form by describing Lasthénie de Ferjol Syndrome, a self-induced iron-deficiency anemia caused by surreptitious bleeding. He named the syndrome after the heroine in Barbey d'Aurevilly's novel, “The Story without a Name” [2,5,6]. In 1977, pediatrician Roy Meadow introduced the term “Munchausen syndrome by proxy” to describe a variation of child abuse where some caregivers consistently fabricate stories and evidence, subjecting their dependents to unnecessary medical investigations and operations [2,3,5,6]. A year later, Wallace and Fitzmorris describe the SHAFT syndrome (Sad, Hostil, Anxious, Frustrating, Tenacious), as a passive variant of MS, where the patient induces the professionals to cause injuries by unnecessary procedures [3].

Over the years, the understanding and classification of factitious disorders evolved, leading to the introduction of specific diagnostic criteria. According to the Diagnostic and Statistical Manual of Mental Disorders, Third Edition (DSM-III), factitious disorder (FD) was officially recognized as a diagnostic category to bridge the gap between hysteria and malingering. It was conceptualized as a condition resulting from deception, driven by internal motivations unrecognized by the individual, manifesting as a commitment to assuming the role of a patient. The disorder could exhibit predominantly psychological, predominantly physical, or both presentations [5,6]. In the latest edition, the DSM-5, FD is categorized within Somatic Symptom and Related Disorders. Two subtypes are distinguished: Factitious Disorder Imposed on Self and Factitious Disorder Imposed on Another (formerly known as MS by proxy) [5,6]. The essential feature of FD involves the falsification of medical or psychological signs and symptoms, either in oneself or another, coupled with identified deceptive behavior [1,5]. The diagnostic criteria demand evidence that the individual is taking actions to distort, mimic, or induce signs or symptoms of illness or injury, devoid of apparent external gains [1-6]. Methods of feigning illness may include exaggeration, fabrication, simulation, or induction. While a pre-existing medical condition might be present, the deceptive behavior or injury induction associated with deception leads others to perceive these individuals (or those influenced by such behaviors) as more ill or disabled than they genuinely are [4,5].

Factitious disorders, particularly MS, entail a risk of excessive clinical interventions, resulting in significant sociofamilial, occupational, and financial impacts [3,7-9]. Individuals with MS often exhibit chronic and extreme patterns, involving multiple hospitalizations, navigating various healthcare units, and seeking unnecessary medical-surgical procedures [1,3,5,7]. Therefore, understanding the evolving diagnostic criteria and the intricate nature of MS is crucial for effective management and a multidisciplinary approach to address the associated challenges.

Through a case report of a patient observed in the context of Liaison Psychiatry, we aim to address MS, considering its characterization, differential diagnoses, therapeutic measures, associated challenges, and the role of Liaison Psychiatry, emphasizing the importance of a multidisciplinary approach of patients.

## Case presentation

We describe a case of a patient observed in a plastic surgery yard within the context of Liaison Psychiatry, following a request for diagnostic clarification. The patient is a 44-year-old married woman and mother of two daughters (25 and 21 years old). She lives with her husband and daughters, has completed the 5th grade, and retired on disability in 2016 due to a complex regional pain syndrome, with lymphedema of the left-hand post-trauma. She previously worked as a cleaning employee in a health center and a forestry company.

Personal history

In terms of personal history, she is the second daughter of a three-sibling family, with an older brother by 4 years and a younger sister by 3 years. She described a more affectionate and close relationship with the older brother, she denied traumatic events in childhood and adolescence and characterized those periods as “very happy” (sic). She also denied significant periods of illness during those life stages. She focused more on her relationship with her mother and only mentioned her father when questioned, responding evasively. After her marriage, she spent a lot of time alone at home as her husband traveled frequently for work.

Psychiatric and medical history

There is vagueness regarding psychiatric history. She claimed to have had a brief follow-up when she “had more pain” (sic) but abandoned the consultation. Currently, there is a record of poor therapeutic adherence. She mentioned ongoing follow-up in private Psychiatry consultation, with the last visit approximately 7 months before the last Liaison Psychiatry observation. She reported being medicated with duloxetine 60 mg/day, clonazepam 2 mg/day, and alprazolam XR 0.5 mg/day, which she claimed to be maintaining. She also recalled a depressive episode after the birth of her youngest daughter, receiving private psychiatric and primary care psychology consultations.

Family history

In terms of family history, notable mentions include a history of breast cancer in the maternal family (grandmother at 80, three aunts at 45) and a maternal uncle with lung cancer at 50. It is noteworthy that the maternal grandmother had both legs amputated and passed away during hospitalization, a difficult loss for the patient as she was an important affective figure. The patient denied significant familial psychiatric history but there is a reference of a father with alcohol use disorder.

Current disease history

The current illness history began in 2008 with the diagnosis of a fibroadenoma in the right breast. She underwent excision of the nodules at Hospital A, but wound healing was complicated, requiring multiple hospital care. In the following year, she started experiencing persistent pain complaints, leading to follow-up at the hospital's pain consultations. She was discharged from the surgery consultations in the same year.

In 2013, she was diagnosed again with multiple small fibroadenomas but did not require further intervention, resulting in discharge from Hospital A's surgery consultations. A few months after discharge, she fell, leading to lymphedema in the left hand and subsequently chronic pain. She sought vascular surgery consultation at Hospital B, underwent vascular exams that were normal, and was then discharged and oriented to physiotherapy.

In 2014, she sought a second opinion at Hospital C, in another city, leading to vascular surgery consultation at Hospital D for surgery. The following year, she underwent thoracic sympathectomy at Hospital D, which, according to the patient, it worsened the edema and pain, posteriorly characterized as complex type II, a neurological disorder that arises after an injury or trauma to a peripheral nerve and can produce long-lasting intense pain, resulting in functional impairment. Until that date, she maintained pain appointments at Hospital A. Due to treatment non-response and subsequent development of depressive symptoms, she was referred to the Psychiatry consultations at Hospital A. There, she was prescribed trazodone OD 300 mg/day, and fluoxetine 20 mg/day.

In 2016, she was hospitalized due to a hand lesion, as it was infected and needed drainage, but the patient requested discharge against medical advice. In the same year, she also abandoned the Psychiatry and Pain consultations at Hospital A and started pain consultations at Hospital B. Afterwards, she got retired and showed improvements in the left upper limb lesion. A few months later, a contact burn on the right foot was documented, with difficult healing and non-compliance to therapeutic recommendations. She then started plastic surgery consultations at Hospital B, leading to her first grafting surgery.

In 2017, the patient had three plastic surgery hospitalizations for grafting, with repeated graft failures after total integration. Additional failure of pain treatments and persistent depressive symptoms led to a referral to Liaison-Psychiatry pain consultations at Hospital B. She had her first appointment in early 2018 and was prescribed trazodone 300 mg/day OD, duloxetine 60 mg/day, with poor adherence to therapeutic measures. During the brief follow-up, MS was considered but not discussed with the patient. However, she abandoned the appointments after the second visit, with multiple consecutive absences. In the same year, she was hospitalized again for another grafting surgery, which again failed.

In 2019, she was also seen in the dermatology consultations at Hospital B for an unspecified dermatosis on the right foot (ulcers with a bizarre appearance on the plantar surface). Biopsies were inconclusive, and FD was considered, leading to discharge. In the same year, during a plastic surgery consultation, it was discovered that the patient was opening the wounds and she was confronted but became offended and did not admit it. She also developed an infectious process in the ulcers. In accordance, in 2020, she was seen by a psychologist at Hospital B due to ongoing pain, showing no motivation for follow-up and therapeutic non-compliance. She eventually discontinued follow-up.

In 2021, she was seen in the Neurology Consultations at Hospital B for complaints of altered sensitivity in the lower limbs, not confirmed, as the patient denied such complaints. Regarding the dermatosis, she had an internal medicine consultation, and autoimmune causes were excluded. In the same year, she was discharged from the vascular surgery consultations, as from a vascular perspective, the lesions showed potential for healing, and she was again referred to physiotherapy.

In 2022, she abandoned the pain consultations at Hospital B and requested an orthopedic consultation at Hospital A. In that consultation, she denied making the request and was later referred to the orthopedic consultations at Hospital B.

**Figure 1:**
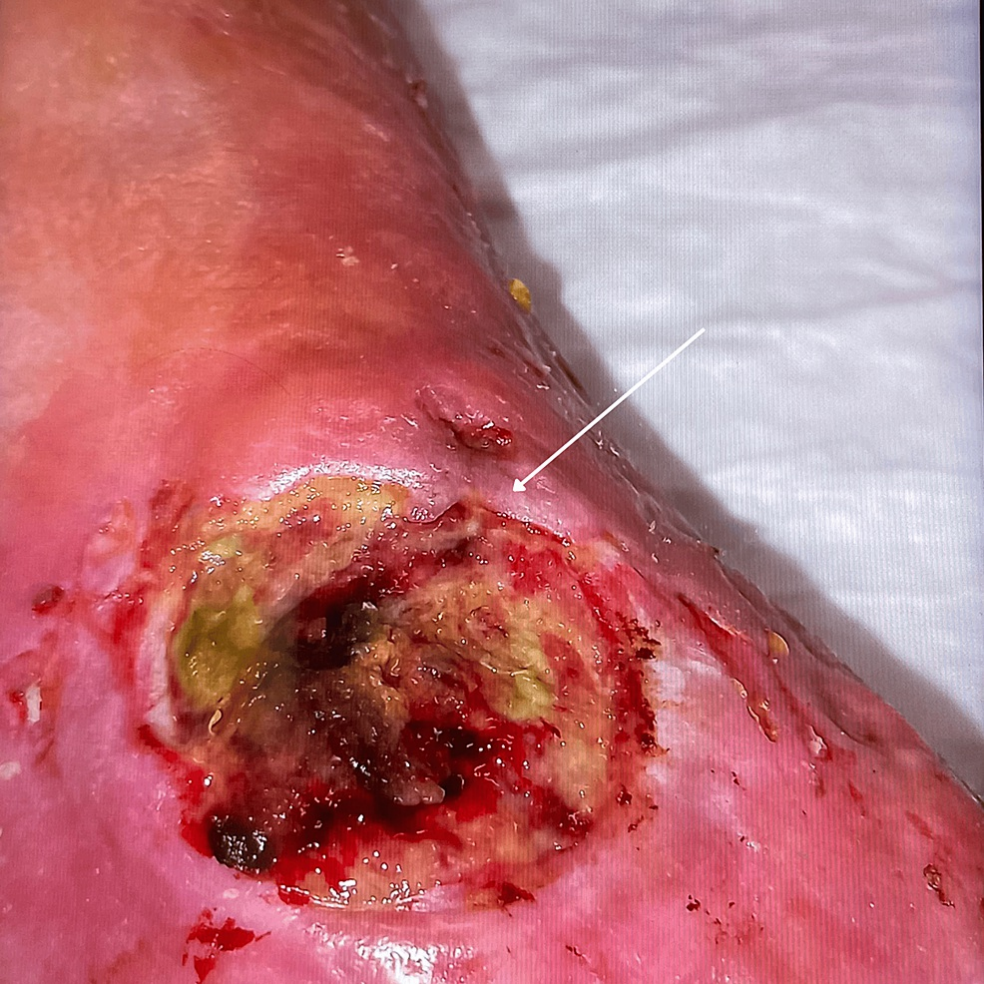
Right tibiae-tarsal cutaneous ulcer

**Figure 2:**
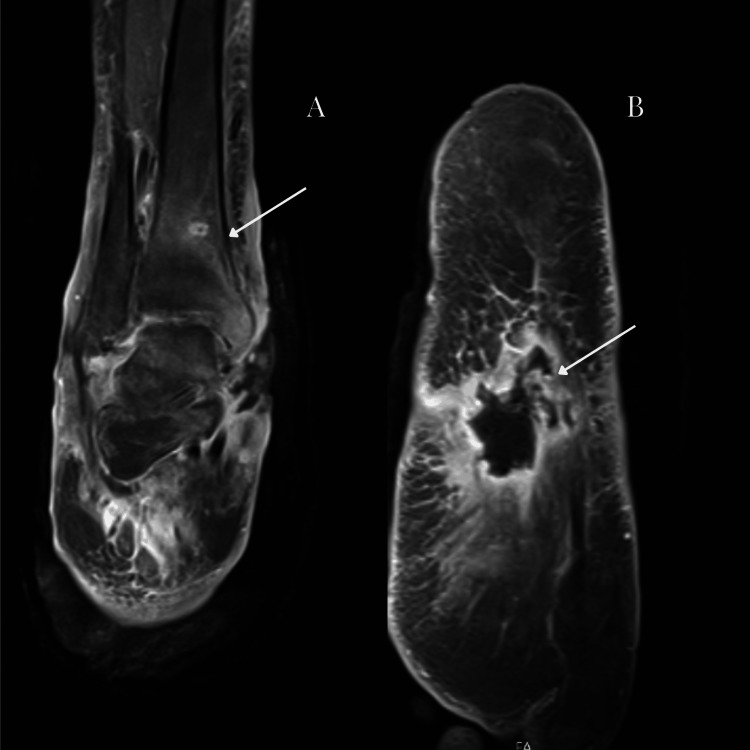
Right foot magnetic resonance imaging (osteomyelitic lesions) A) Area of cutaneous ulceration with a fistulous tract, associated with a pronounced adjacent inflammatory process, accompanied by thickening of the posterior tibial tendon and erosion at the medial region level, B) Dysmorphia with ankylosis and erosions of the bony elements of the midfoot, in relation to changes in the context of chronic osteomyelitis

In 2023, she received a diagnosis of chronic osteomyelitis of the right lower limb, involving the foot and tibiae-tarsal joint, with cutaneous ulcers (Figure 1), confirmed by foot magnetic resonance imaging (Figure 2). She was then proposed for amputation and was admitted for the eighth time for plastic surgery. It was during this hospitalization that collaboration with Liaison Psychiatry was requested.

During the first Liaison Psychiatry observation, the patient exhibited appropriate contact most of the time, responded to questions, and dramatically recalled the time when she had “breast cancer” (sic), claiming to have undergone various treatments, including chemotherapy, with associated hair loss, which did not happen in reality. She described the evolution of her clinical condition up to the present moment and deliberately showed pictures of the lesions. She expressed hopefulness about the possibility of amputation, stating, “I prefer amputation to remaining like this (…) this foot is no longer useful” (sic), and expressed anxiety only about the possibility of it not happening. Despite limitations, she described a functional daily life, with good support from close family members, a very supportive husband, and always available daughters. Since complications in the healing of leg lesions, she started accompanying her husband on his travels. She proudly mentioned that her younger daughter had been depressed in response to her clinical situation, and her older daughter, who was soon to be married, would be residing in her house to provide more support. She also described support from the extended family. She admitted being sadder and more hopeless when she had more pain. During the interview, she focused on nearly total insomnia persisting for several months. Despite this, she did not seek help in Psychiatry consultation again. She said she was very angry when they suggested that she might be causing injuries to herself, stating, “well, why would I do that to myself(...)go through all this” (sic). On the mental state examination, she appeared with belle indifference, neutral mood, minimal affective resonance, no identifiable heterologous activity, and preserved instinct for self-preservation. Thus, therapeutic adjustments were made, reintroducing duloxetine 60 mg and trazodone 100 mg.

During the hospitalization, she remained stable hemodynamically. An occlusive dressing was applied to prevent manipulation of the lesions by the patient. She complained of intolerance to the proposed treatments, especially those most effective for ulcer healing. A multidisciplinary meeting was held, including plastic surgery, orthopedics, physical medicine and rehabilitation, Liaison Psychiatry, and the patient's husband. Treatment and post-surgical follow-up guidelines were established. During the meeting, the husband was also informed of the probable diagnosis, he seemed to understand, although considering it unlikely, but did not rule out the possibility, pointing out that the wife spent a significant part of the day alone without any supervision. On the same day, right transtibial amputation was performed due to ulcers with poor evolution and associated osteomyelitis. The procedure went without complications and the postoperative recovery was without issues. Upon discharge, the patient reported feeling “very happy and satisfied with the surgery, with no complaints.”

According to Liaison Psychiatry, the patient was recommended for follow-up during hospitalization, in the postoperative period, and for consultation follow-up after discharge. Family members were involved in promoting the patient's adherence to the recovery and rehabilitation process. To date, she has shown poor compliance with psychiatric follow-up. Moreover, there is a probable recurrence of self-injurious behavior, sustained by the development of an unexpected abscess in the amputation stump and difficult healing.

## Discussion

The clinical case presented highlights the intricate interplay between chronic medical conditions, psychiatric factors, and challenges in diagnosing and managing of MS. It also emphasizes the importance of a multidisciplinary approach.

As previously mentioned, MS is the severe form of FD and can be distinguished from other FDs by the following characteristics: the chronic and severe nature of the factitious illness behavior; intentional production of signs and symptoms through medically dangerous manipulations of the patient's body, ensuring prolonged hospitalization; the observed hospital-peregrination behavior, wherein the patient moves from one facility to another, often across cities or countries, seeking new care upon the fear of exposure; and the presence of pseudologia fantastica, where the patient makes false claims about accomplishments, educational credentials, or relationships [1,3,5,7]. These characteristics are evident in the presented case, where the patient dramatically reported having breast cancer when, in reality, only had benign lesions. The hospital-peregrination pattern was also observed as the patient visited four different hospitals over the years of illness and the severe nature of self-inflicted lesions, often leading to invasive procedures.

Although severe and widely described, the prevalence of FD/MS is poorly understood for various reasons, including the variable and complex presentations that are often challenging to distinguish from genuine medical conditions, frequent hospital escapes or discharge against medical advice before identification, and the reliance on case reports and limited case series in the literature with insufficient long-term follow-up data [1,3,7,10]. It is estimated that FD is present in approximately 1.3% of all hospitalized patients, with MS accounting for about 10% of cases [6,7,9,11,12] and the highest estimate given by a dermatologist and neurologist [6]. But it can be found in all medical specialties, as described by Yates and Feldman (2016) in their systematic review, including 455 cases of FD [12]. Despite its relatively low recognized prevalence, MS is associated with significant morbidity, healthcare costs, and even mortality [6,7].

Evans et al. (2021) conducted a systematic review involving 42 cases of FD in plastic surgery. The review revealed that 66% were women, 62% worked in the healthcare sector, 70% had comorbid psychiatric conditions (50% depression), and 93% had self-inflicted injuries. The hand was the preferred target for self-inflicted cutaneous injuries, with an average delay in FD diagnosis of 54 months. Approximately 46% of patients underwent multiple surgical procedures during this period. Surgical wounds were often explored or manipulated by patients, and 50% contaminated or manipulated the wounds to prevent healing. Notably, 36% resulted in significant long-term disability, and 10% of patients underwent amputation. These findings closely align with the observed case [10]. As it can be seen, the patient described in this report match these characteristics.

Risk factors and etiopathogenesis

Risk factors for FD, including MS, remain poorly understood [1,3,7,13]. Based on reported cases, certain predisposing factors include a history of psychiatric or medical conditions in childhood or adolescence that garnered marked medical and family attention, a history of personality disorders, especially borderline, narcissistic, or antisocial personality disorders, employment in the healthcare sector, female gender (aged 30-40 years), and dissatisfaction or resentment toward the medical profession or a significant past relationship with a medical professional [1,3,7,13].

The etiopathogenesis of FD is unknown, with no identified structural or functional brain abnormalities [3,14,15]. Some patients exhibit abnormalities in psychological tests, particularly dysfunctional personality traits, including impulse control difficulties, and self-destructive or passive-aggressive behavior [4,3,15]. Despite these observations, different theories attempt to explain the etiopathogenesis of FD [4,6]. It is consensual that a primary motivator could be the appeasement of the need for attention, acting, and being treated as sick or incapacitated-termed “sick role” [1-4,6,14]. Some patients may derive pleasure from challenging and deceiving healthcare professionals, aligning with psychopathic tendencies [1,3]. Adopting the “sick role” provides acceptance and unconditional care, and hospital admission confirms the “sick role” within the social network. Studies describe that with the “sick role”, patients can satisfy unmet psychological needs for support, compassion, and sympathetic understanding [1,4].

A study performed by Galli et al. (2017) also supports the role of social learning mechanisms in FD behavior [6]. According to this study, many FD patients personally experienced a severe illness in childhood or had a family member who did [6]. These experiences introduced the child to the various benefits associated with the sick role, reinforced by positive attention or avoidance of responsibilities, predisposing individuals to develop FD behavior with underlying psychological vulnerabilities [6]. The literature also describes an association between the development of FD/MS and childhood trauma (sexual abuse, physical, emotional, or psychological violence), a history of neglect or situations of emotional deprivation in childhood, and the loss of significant attachment figures at an early age, especially in cases of death from illness [13,16]. This is evident in the presented case, where the patient experienced neoplastic illness in the family, her own fibroadenoma, and the amputation of her grandmother's limb, who was a significant emotional figure, during hospitalization leading to her demise. We also hypothesize that the patient had a harsh childhood, taking into account having an alcoholic father whom she barely mentioned. This could also explain the dysfunctional personality traits and behaviors.

Diagnosis

Regarding the diagnosis, studies emphasize the importance of determining whether it is a single episode of MS or recurrent, following basic procedures to respond to apparent signs and symptoms, understanding the reliability and validity of medical tests performed, and respecting base rate information on the prevalence of various diseases that should be ruled out [3,15]. It is consensual that the diagnosis of FD (including MS) relies on evidence of intentional symptom production in the absence of an objective reward associated with the behavior [1,3,14,15]. However, probable diagnostic elements have been described, as illustrated in Table 1 [2,3,7,14]. In the case presented, the patient exhibits all elements, including a history of multiple admissions to different hospitals and various treatment interventions, as well as paradoxical enthusiasm for surgical treatments and invasive procedures.

**Table 1:** Factors contributing to a probable diagnosis of MS MS: Munchausen Syndrome

Factors contributing to a probable diagnosis of MS
Young female patient
Work in a healthcare service provider setting
Familiarity with medical terminology and hospital procedures
History of psychiatric illness
History of multiple admissions to various hospitals and numerous treatment interventions
Dramatic, vague, and inconsistent clinical history
Atypical clinical presentations of the symptoms
Familiarity with medical terminology and hospital procedures
Inappropriate disruptive behavior in the ward (arguments with nurses and doctors, non-compliance with rules)
Paradoxical enthusiasm for surgical treatments and invasive interventions
Rapid development of “complications” or a new illness if the evaluation does not reveal issues

According to the literature, in the mental state examination, possible findings include an attitude ranging from anxious evaluation and/or invasive treatment to an evasive and vague attitude toward details, mood and affect incongruent with what would be expected based on the patient's medical condition, and an “artistic Mona Lisa” expression of innocent indifference (belle indifference) [14,15,17]. In FD with predominantly psychological signs and symptoms, abnormalities in perception, suicidal or homicidal tendencies, or aberrant cognitive functioning, especially in orientation and recall, are described [4,15]. Psychopathology compatible with the presence of psychiatric comorbidities, often borderline personality disorder and/or antisocial personality disorder, and depressive disorder may also be present [3,4,14,15].

Differential diagnosis

The broad differential diagnosis of MS includes not only organic conditions but also somatic symptom disorders, malingering, conversion disorder, and borderline personality disorder (Tables 2-3). Distinguishing features include self-inflicted harm associated with deceptive behavior and the absence of obvious secondary gain [2,3,15,16]. Differential diagnosis is also important with schizophrenia with cenesthesis hallucinations and mood disorders (depression) [9,11,15]. As many patients appear functional and productive outside the hospital environment, detection, even by close caregivers, remains challenging [9,15]. Unfortunately, some self-injurious behaviors can lead to genuine illness over time, while others are more episodic but still potentially life-threatening [3].

**Table 2:** Main differential diagnosis

Disorder	Mechanism of disease production	Motivation of illness behavior	Main goal	Presence of recognized gain
Functional neurological symptom disorder	Unconscious	Unconscious	Not identifiable	No
Factitious disorder	Conscious (intentional)	Unconscious	Deception to assume the sick role	No
Malingering	Conscious (intentional)	Conscious	Specific goal in mind (financial compensation, avoiding legal consequences, stopping work)	Yes. Symptoms improve/disappear when they cease to be useful.
Borderline personality disorder	Conscious (intentional)	Conscious	Emotional regulation (no associated deception)	Yes

**Table 3:** Differential diagnosis between FD and Malingering FD: factitious disorder

Malingering	Factitious disorder
Male > female	Female > male
Substance abuse	Employment/training in the medical field
Vague and inconsistent medical history	Vague and inconsistent medical history
Refuses invasive tests and procedures	Enthusiasm for more invasive tests and procedures
Predominant comorbidity with antisocial personality disorder	Predominant comorbidity with borderline personality disorder

Challenges in diagnosis

Alert signs can be applied to patients with non-factitious chronic illnesses. For example, these chronic patients may also exhibit high healthcare service utilization, normal test results, atypical presentations, or treatment failure [6,14,16]. Physicians may avoid diagnosing FD when a symptom is challenging to explain, and when deceptive behavior has not been formally established, it may seem less risky for a doctor to conclude an unknown origin disease instead of FD, especially because doctors are not always familiar with the diagnosis [5,14,16,17]. Moreover, there is considerable stigma associated with the diagnosis [15]. Therefore, a definitive diagnosis requires confession or evidence of deceptive behavior [1,6], demanding a paradigm shift, a more comprehensive view of the patient, and a greater focus on multidisciplinary approaches considering its complexity.

On the other hand, Lawlor and Kirakowski (2014), on their grounded theory analysis of an online support group for FD, challenged the common perception that motivation for FD is unconscious, finding that FD sufferers are conscious of their motivations. Contrary to conventional views, the analysis revealed that FD sufferers do experience internal symptoms associated with the disorder and are upset by their behavior. They also found that FD sufferers were deterred from seeking formal help by anticipated fears and instead opted for self-management strategies and common barriers to seeking help included fears of disclosure, loss of family/friends, confronting issues, logistical difficulties, fear of recovery, and fear of perpetuating FD. The study hypothesized that the characteristics of FD described by the group members were congruent with those associated with addiction, suggesting a potential link between FD and addictive behavior [18]. These assumptions need further investigation.

Treatment

Similar to the diagnostic process, caring for patients with FD/MS is highly challenging. People with MS often elicit some form of repulsion from the medical team, exacerbating the difficulty in treatment [3]. The diagnosis is rarely accepted by patients, and none admit to attempting to deceive others [3,9,14]. Therefore, the primary treatment goal should be to reduce the number of potentially harmful tests and surgical procedures and establish care primarily focused on psychiatric aspects [3,9,13,14,17]. Treatment should be multidisciplinary, invariably involving Psychiatry [1,3,5,13-15,19]. Preserving the doctor-patient relationship is crucial to prevent further hospital-peregrination, and specific appropriate treatment for each type of MS should be provided, involving the patient in their therapeutic process [3,9,15]. In this context, it is essential to remember that an individual with MS may not comprehend the logic behind their diagnosis and may not believe they suffer from the disorder [14,15].

While no specific therapy has proven effective, the focus should be on behavioral and symptomatic control rather than cure, without diverting attention from the underlying emotional suffering [1,9,15]. Therefore, in the presence of suspected MS, referral for psychiatric evaluation is essential [1,3,9,15,19]. But, even with a higher probability of MS, standard care should be provided, including necessary surgical care for any condition and comorbid complications [15].

The role of Liaison Psychiatry

As previously mentioned, psychiatric intervention is crucial [9,10,13-15,17]. Liaison Psychiatry is essential for establishing the diagnosis, identifying associated comorbidities, establishing a follow-up and treatment regimen, and providing education to the medical team about the condition [10,15,17]. Once the diagnosis is confirmed, addressing the underlying emotional needs of the patient to determine the motivation for MS is important [3,15,17].

The treatment priority focuses on enabling the individual to recognize when compelled to engage in factitious illness behavior and preventing its recurrence [3,15,14]. It is also advisable to conduct an interview with the patient and at least two other individuals [3,13,14].

The psychiatric management of MS should be a long-term process, typically involving the following steps: recognizing and gaining a better understanding of the problem; developing more effective coping strategies; increasing empathy toward individuals negatively impacted by the fabrications (friends, family, professionals); taking responsibility for one's own recovery; and developing a helpful and adaptive support system [1,3,14]. Treating psychiatric comorbidities is essential, as well as addressing present psychiatric symptoms [3,14].

Some authors advocate for a confrontation strategy [6,13]. However, confrontation should be done empathetically and tolerantly, informing the patient about the team's suspicions and the evidence supporting them [6,13,15]. The medical team should maintain a compassionate attitude during the confrontation and throughout the entire treatment [15]. Expressing empathy for the suffering the patient must have experienced or the stress under which they were, resulting in illness-causing behaviors, is fundamental [6,13,15]. Prioritizing comments that express concern for the patient's ongoing health and well-being and the desire to continue caring for them can be helpful in maintaining the relationship [15]. It is important to note that a patient confronted with suspicions that their illness is factitious may not be receptive to education attempts, as observed in our patient's case, where confrontation served to further fuel the behavior, possibly due to not being conducted by a professional trained for such situations.

In addition to confrontation, educating the patient is necessary. This education should be attempted in the same gentle, tolerant, and supportive manner. If the patient grants permission, educating family members about the patient's condition can also be useful [15]. Education about the risks of non-compliance with treatment recommendations is also important, ethically and legally, as the patient may want to leave the hospital against medical advice [15].

Patients are markedly resistant to psychiatric evaluation, and information is often difficult to obtain, as observed in the case of the described patient, who was vague in describing her history [3,9,14,16]. Therefore, it is essential that the responsible medical team explains to the patient the concerns about the possibility of the condition recurring without adequate follow-up [15]. In this process, the liaison psychiatrist should be introduced to the patient as an integral part of the responsible medical team, promoting greater adherence [15].

In addition to follow-up with Liaison Psychiatry consultations, the patient may benefit from individual supportive psychotherapy [6,15]. However, there is little information available on the subject, likely due to poor patient adherence. Studies suggest approaches such as family therapy and cognitive-behavioral therapy [15]. Less conventional therapies such as hypnotherapy and mindfulness-based therapies are also suggested as potentially helpful approaches for the management of individuals with FD [6].

However, a systematic review by Eastwood and Bisson (2008), concluded that there was no discernible distinction in outcomes when comparing the utilization of the confrontation approach, psychotherapy, and psychiatric medication versus non-utilization. The review suggested establishing a centralized registry of reports to enhance the development of evidence-based guidelines [20].

Prognosis

The progression of MS may involve only a few short episodes but generally becomes chronic, making the prognosis guarded [3,9]. Studies demonstrate the importance of maintaining contact with the patient once the diagnosis is made to prevent hospital-hopping and a new cycle of illness [3,13]. Although treatment may temporarily improve symptoms, it does not seem to endure in most cases, although cases of individuals who recovered are described [15]. In our case, the recurrent and chronic nature of MS is evident.

Another prognostic factor is the presence of a treatable concurrent psychiatric illness, such as major depression, which is considered a sign of a good prognosis [3]. Additionally, some researchers believe that FDs attenuate with age and maturity, as is the case with personality disorders [3,9,15]. It seems that, even without treatment, the simple form of FD sometimes improves in the fourth decade of life [15]. As also observed in the described case, the factitious production of illnesses can lead to emotional suffering for the patient and their family [9,15].

Elwyn et al. (2019) described some characteristics that increase morbidity and mortality in MS cases, namely: patients engage in dangerous manipulations of their bodies that can also result in unintentional severe injuries, permanent disability, or death; patients incur a substantial risk of iatrogenic diseases and injuries by repeatedly engaging in deceptions that lead healthcare providers to perform risky diagnostic procedures and treatments; patients often provide incomplete or false information about medical history that, intentionally or inadvertently, causes increased morbidity or mortality [15].

Finally, as patients with MS undergo various medical procedures, the lifetime risk of experiencing an unintentional adverse medical event is often higher than that of the general population [9].

## Conclusions

Feigning symptoms can be seen as a manifestation of a deeper, profound pain that necessitates appropriate action from healthcare professionals. As observed, the diagnosis and treatment of MS pose challenges, with a delicate balance between accurate diagnosis, diagnostic errors, and underdiagnosis. On the other hand, early diagnosis is crucial, aiding in the prevention and treatment of mental and physical consequences that may lead to premature death and significant disability. Moreover, MS should be considered in the differential diagnosis when the disease presentation is atypical, or an underlying cause cannot be identified.

Increasingly, a multidisciplinary team approach is recommended to streamline both diagnosis and treatment. Early involvement of Liaison Psychiatry should be encouraged, even though patients often decline it, and psychiatric comorbidities must be identified and promptly addressed. While early detection and diagnosis may not alter the course of the disease in MS patients, they can prevent problematic forensic issues, healthcare and familial costs, and invasive and potentially lethal iatrogenic interventions. Therefore, there is a need for heightened awareness and suspicion of MS, coupled with a reduction in associated stigma that could delay diagnosis, act as a barrier, and hinder patients from receiving appropriate treatment. Additionally, continuous psychiatric support and family involvement are critical in addressing the recurrence of self-injurious behaviors. Further research is imperative to enhance understanding of MS, as well as contribute to an improved approach to diagnosis, intervention, and ultimately, the overall management of this challenging psychiatric disorder.
